# Are changes in sleep problems associated with changes in life satisfaction during the retirement transition?

**DOI:** 10.1007/s10433-024-00802-4

**Published:** 2024-03-12

**Authors:** Marika Kontturi, Marianna Virtanen, Saana Myllyntausta, K. C. Prakash, Jaana Pentti, Jussi Vahtera, Sari Stenholm

**Affiliations:** 1 School of Educational Sciences and Psychology, University of Eastern Finland, P.O. Box 111, 80101 Joensuu, Finland; 2 Division of Insurance Medicine, Department of Clinical Neuroscience, Karolinska Institutet, Stockholm, Sweden; 3 Department of Psychology and Speech-Language Pathology, Faculty of Social Sciences, University of Turku, Turku, Finland; 4 Unit of Health Sciences, Faculty of Social Sciences, Tampere University, Tampere, Finland; 5 Department of Public Health, University of Turku and Turku University Hospital, Turku, Finland; 6 Clinicum, Faculty of Medicine, University of Helsinki, Helsinki, Finland; 7 Centre for Population Health Research, University of Turku and Turku University Hospital, Turku, Finland

**Keywords:** Sleep problems, Insomnia, Sleep quality, Life satisfaction, Retirement, Retirement transition

## Abstract

Retirement reduces sleep problems, but changes in life satisfaction during the retirement transition are multifactorial and partly unknown. The aim of this prospective cohort study was to examine whether changes in sleep problems are associated with changes in total and domain-specific life satisfaction during the retirement transition (on average 0.5 years before and 0.5 years after retirement). The study population consisted of Finnish public sector employees (*n* = 3518) from the Finnish Retirement and Aging (FIREA) study who responded to annual surveys before and after transition to statutory retirement. Sleep problems were measured with Jenkins Sleep Problem Scale questionnaire and participants were grouped into four sleep problem groups depending on the state of their sleep problems during the retirement transition: ‘Never,’ ‘Decreasing,’ ‘Increasing,’ and ‘Constant’ sleep problems. Life satisfaction was measured with the Life Satisfaction Scale questionnaire including four domains (interestingness, happiness, easiness, togetherness). We found that the improvement in total life satisfaction was greatest for participants in the ‘Decreasing’ (0.17, 95% CI 0.11–0.23, SMD 0.27) and ‘Constant’ (0.12, 95% CI 0.07–0.18, SMD 0.19) sleep problem groups. Of the specific life satisfaction domains, similar findings were observed only for the easiness domain. It seems that decreasing or constant sleep problems are associated with improved life satisfaction during the retirement transition, especially in the feeling of easiness of life. This may be due to the fact that as the demands of working life are removed, sleep problems are alleviated or it becomes easier to live with them, which improves life satisfaction.

**Supplementary Information:**

The online version contains supplementary material available at 10.1007/s10433-024-00802-4.

## Introduction

Retirement is one of the most important life transitions in late midlife. People adapt to retirement in different ways, and the adaptation can be either a short- or a long-term process (Wetzel et al. 2016). The adaptation depends on many personal characteristics, such as the retiree’s sex. Additionally, it depends on many situational characteristics, such as context of retirement, which means, for example, the type of pension, and other life events in the retiree’s life (Henning et al. 2016). Thus, along with the formal, objective life course transition, retirement is also a social psychological transition that is related to psychological well-being (Moen 2001).

Sleep quality is an important component of subjective well-being (Paunio et al. 2008; Piper 2016), and it may also change during the retirement process, because transitioning from work to retirement alters the typical daily routine for adults (Eibich 2015). The previous cohort studies have shown that sleep problems are common during the final working years and that sleep improves following retirement, in terms of both longer sleep duration (Eibich 2015; Myllyntausta et al. 2017; Garefelt et al. 2021), and better sleep quality (Vahtera et al. 2009; van de Straat et al. 2020). Especially, premature awakening and non-restorative sleep have been found to improve after retirement (Myllyntausta et al. 2018).

Life satisfaction is a key indicator of subjective well-being (Erdogan et al. 2012) and it may be viewed as a cognitive evaluation of one’s life according to subjectively determined standards (Diener et al. 1999; Schimmack et al. 2002). Life satisfaction is influenced by a wide range of aspects of life. For example, higher socioeconomic status (Moreno-Agostino et al. 2021), physical activity (Stenlund et al. 2021), and being married or cohabited (Wang 2007) have been positively related to life satisfaction. On the other hand, smoking (Stenlund et al. 2021), obesity (Wadsworth and Pendergast 2014), being a male (Piper 2016), and different negative life events (Luhmann et al. 2012) have been negatively related to life satisfaction. An U-shape has been found between age and life satisfaction (Piper 2016).

Additionally, the retirement transition may affect life satisfaction, but previous findings regarding changes in life satisfaction during the retirement transition are mixed (Kim and Moen 2002; Wang 2007). Most of the previous studies have shown a positive effect of retirement on life satisfaction (Hershey and Henkens 2014; Gorry et al. 2018; Prakash et al. 2022), but some studies have identified a negative effect (Dave et al. 2008) or no effect at all (Abolhassani and Alessie 2013).

The inconsistency of the study results may partly be due to life satisfaction’s multifactorial nature, as people may be satisfied with different components of their life. Most of the previous studies among older people have assessed satisfaction with one’s life in general, while the specific domains of life satisfaction have received less attention. However, previous studies have shown that some domains of life satisfaction have a substantially larger influence on health and well-being outcomes than others (Nakamura et al. 2022). To the best of our knowledge, only three studies have considered the domain-specific life satisfaction during the retirement transition (Bonsang and Klein 2012; Calasanti et al. 2021; Prakash et al. 2022). Bonsang and Klein (2012) found increased satisfaction with leisure time but decreased satisfaction with household income among men during the retirement transition. Calasanti et al. (2021) showed that only men reported increased life satisfaction during the retirement transition, particularly in financial satisfaction. Prakash et al. (2022) found the greatest improvement in the easiness domain of life satisfaction during the retirement transition.

The studies that have examined the association between sleep quality and life satisfaction have shown that better sleep quality and greater life satisfaction are associated with each other (Ness and Saksvik-Lehouillier 2018; Papi and Cheraghi 2021; Zhi et al. 2016). However, these studies have mostly been cross-sectional in nature. The few longitudinal studies have shown that better sleep predicts greater life satisfaction in young adults (Shin and Kim 2018) or people of all ages (Paunio et al. 2008), but life dissatisfaction does not consistently predict poor sleep (Paunio et al. 2008). However, reciprocal effects have been reported between sleep quality and life satisfaction in older adults (Zhu et al. 2023). Furthermore, although the findings show that life satisfaction increases when sleep quality increases in general (Kudrnáčová and Kudrnáč 2023), we are not aware whether these changes are interdependent during the retirement transition. There are also no studies on whether changes in sleep are associated with changes in different domains of life satisfaction.

To fill the gaps of the existing literature, the aim of this prospective cohort study was to examine how changes in sleep problems are associated with changes in life satisfaction during the retirement transition. To have a more specific understanding of the association between sleep and life satisfaction, we assessed both the total and domain-specific life satisfaction.

## Methods

### Study design and population

To obtain the analytical sample, we used the questionnaire data for the analysis, and we centered the data around the actual retirement date, obtained from the questionnaires. There were two possible study waves before retirement (wave − 2, wave − 1) and three possible study waves after retirement (wave + 1, wave + 2, wave + 3), with each successive wave one year apart from each other. We specifically wanted to study the association between changes in sleep problems and life satisfaction in a narrow time window, so we focused the main analyses on just before and after the transition to retirement (wave − 1 to wave + 1). The study design is presented in Online Supplementary Table 1.

The study population consisted of participants of the Finnish Retirement and Aging (FIREA) study which is an ongoing prospective cohort study of older adults in Finland established in 2013 (Stenholm et al. 2023). For this study, we included participants who had answered the Jenkins Sleep Problem Scale questionnaire and Life Satisfaction Scale questionnaire in at least two surveys, one immediately before and one immediately after the transition to statutory retirement (*n* = 3518). The selected participants provided information on life satisfaction at an average of 3.8 (SD 0.6) study waves.

The aim of the FIREA study is to study changes in health behavioral and cardiometabolic risk markers across retirement transition, and to examine the long-term consequences of work and retirement on health and functioning with advancing age (Stenholm et al. 2023). The eligible population (*n* = 10,629) for the FIREA study cohort included all public sector employees whose individual pensionable age was set between 2014 and 2019 and who were working either in one of the 27 municipalities in Southwest Finland or in one of the selected nine cities or five hospital districts around Finland in 2012. Participants were first contacted 18 months prior to their estimated retirement date, which was obtained from the pension insurance institute for the public sector in Finland (Keva). The pensionable age is individual due to, for example, the year of birth of the person. Thereafter, the participants have been followed up with annual surveys up to seven times (mean 3.6, SD 1.4 surveys), with an aim to gather data from at least two time points before and two time points after the transition to statutory retirement. Of the FIREA cohort members, 6783 (response rate 64%) had responded to at least one survey by the end of December 2018. Of these respondents, 5195 were those who answered for the first time while still working. The differences between the study population and the FIREA study eligible sample and survey responders are presented in Online Supplementary Table 2.

The FIREA Study was conducted in line with the Declaration of Helsinki and it was approved by the Ethics Committee of Hospital District of Southwest Finland. The participants are volunteers who have given written informed consent to participate in the study. For the analyses, anonymized data were used.

### Study context: timing of retirement in Finland

In Finland, the Public Sector Pensions Act regulates the pensionable age of the public sector employees. From 2005 to 2016, the individual pensionable age in the public sector was generally 63–68 years. Subsequent to the pension reform initiated in January 2017, the minimum and maximum ages for mandatory retirement slightly rose based on birth year and life expectancy. The individual pensionable age is flexible, which means that the pension may be taken out within a certain age range and there is also an upper limit for how long a person can continue working (Finnish Centre for Pensions 2022). In addition to the general retirement age, some public sector employees may still have occupational retirement ages that are lower than the general retirement age (Keva 2023).

### Measures

#### Sleep problems

Sleep problems were assessed with the Jenkins Sleep Problems Scale questionnaire (Jenkins et al. 1988), which includes four items: difficulties falling asleep, difficulties maintaining sleep during the night, waking up too early in the morning, and non-restorative sleep. Participants were asked to estimate how often each of these difficulties had occurred during the past 4 weeks (never, 1–3 nights per month, one night per week, 2–4 nights per week, 5–6 nights per week, or almost every night). If the frequency of the most frequent symptom the participant reported was higher than four nights per week, the participant was considered to have sleep difficulties (yes vs. no), as previously (Salo et al. 2014; Myllyntausta et al. 2018).

Based on participants’ responses to the sleep problem questions in study wave − 1 and study wave + 1, four groups of sleep problems were created. ‘Never’ group indicates participants not having sleep problems neither at wave − 1 nor at wave + 1. ‘Decreasing’ group indicates participants having sleep problems at wave − 1 but not at wave + 1. ‘Increasing’ group indicates participants not having sleep problems at wave − 1 but having sleep problems at wave + 1. ‘Constant’ group indicates participants having sleep problems both at wave − 1 and wave + 1.

#### Life satisfaction

Life satisfaction was measured in each study wave using a four-item Life Satisfaction Scale questionnaire (Andrews and Withey 1976), which was modified from the questionnaire developed to measure the quality of life (Allardt 1973). The Life Satisfaction Scale questionnaire comprises of four domains of life satisfaction, which were inquired with a single question each, namely ‘interestingness’ (‘Do you feel that your life at present is interesting?’), ‘happiness’ (‘Do you feel that your life at present is happy?’), ‘easiness’ (‘Do you feel that your life at present is easy?’), and ‘loneliness’ (‘Do you feel that at present you are lonely?’).

There were four or five response alternatives, and the item responses ‘I cannot say’ were scored as 3 following the procedure used in previous studies (Koivumaa-Honkanen et al. 2000; Prakash et al. 2022). The total life satisfaction score was calculated by averaging the responses of the four domains of life satisfaction. The sum score ranged from 1 to 5 with increasing values indicating better life satisfaction. In addition to the total life satisfaction value, we also calculated the values for each separate domain of life satisfaction by averaging the responses of each domain. To harmonize the scores used in our study, the score of the domain ‘loneliness’ was reversed and renamed to ‘togetherness’ henceforth, as previously (Prakash et al. 2022). If the response was missing for all four domains, the sum score of total life satisfaction was treated as missing.

#### Covariates

Covariates included socio-demographic factors (sex, age, occupational status, and marital status), lifestyle factors (body mass index, physical activity, and smoking), and recent life events (events during the past year) associated with life satisfaction in previous studies (Wang 2007; Luhmann et al. 2012; Wadsworth and Pendergast 2014; Piper 2016; Moreno-Agostino et al. 2021; Stenlund et al. 2021). For descriptive statistics, all covariates were defined at wave − 1, immediately before retirement, except the life events, which were defined at wave + 1, immediately after retirement.

Information on sex and birth year (transformed to age) was obtained from the pension insurance institute for the public sector in Finland (Keva). The occupational titles were obtained from employers’ records, and they were coded according to the International Standard Classification of Occupations (ISCO) 2001 and categorized into three groups: upper-grade nonmanual workers (ISCO classes 1–2, e.g., teachers), lower-grade nonmanual workers (ISCO classes 3–4, e.g., technicians), and manual workers (ISCO classes 5–9, e.g., cleaners) (Statistics Finland 2001).

All the other covariates were based on the survey responses. Body mass index (BMI, kg/m^2^) was calculated based on self-reported body weight and height, and BMI ≥ 30 kg/m^2^ was denoted as obesity (yes vs. no) (World Health Organization 2001). Physical activity was assessed by asking participants to estimate their average weekly hours of leisure time physical activity (including commuting) during previous year in walking, brisk walking, jogging, and running, or their equivalent activities. Weekly physical activity was expressed in metabolic equivalent (MET) hours which was dichotomized into two groups with < 14 MET hours/week defined as low physical activity (yes vs. no) (Kujala et al. 1998; Leskinen et al. 2018). Smoking was assessed by asking whether the respondent currently smoked or had ever smoked and dichotomized into current smoker vs. non-smoker (including never and ex-smokers).

Life events were assessed by asking whether the respondent had experienced some of the listed adverse life events (divorce or judicial separation, worsening of the economic situation, illness or death of a loved one, experiencing violence) during past 12 months, and dichotomized into two groups (yes or no life events).

### Statistical analyses

Characteristics of the participants at wave − 1 were analyzed by frequencies and percentages for categorical, and mean and standard deviation (SD) for a continuous variable (age). Characteristics of the participants were also reported by the four sleep problem groups, and by the mean of total and domain-specific life satisfaction scores. For descriptive statistics, we used the *χ*^2^-test for the association between categorical characteristics and sleep problem groups and the analysis of variance (ANOVA) for the association between continuous characteristics (age) and sleep problem groups and for the association between categorical characteristics and life satisfaction scores. Additionally, a Pearson correlation coefficient was used to examine the association between continuous characteristic (age) and life satisfaction scores.

Secondly, to illustrate the level of total and domain-specific life satisfaction across the entire retirement transition by the four sleep problem groups, we first calculated the mean estimates and their 95% confidence intervals (CIs) for each of the study waves (− 2, − 1, + 1, + 2, + 3). These results are presented as line graphs across the retirement transition (wave − 2 to wave + 3) with an unadjusted *p* value for the changes in total and domain-specific life satisfaction during the retirement transition (wave − 1 to wave + 1).

We examined the association between sleep groups and life satisfaction at pre-retirement (wave − 1) by using multiple linear regression analyses. Mean estimates and their 95% CIs of total and domain-specific life satisfaction by four sleep problem groups are presented to describe the baseline relationships. The estimates and *p* values are given for the comparison of the other sleep problem groups with the ‘Never’ sleep problem group.

We studied the association between changes in sleep problems and changes in total and domain-specific life satisfaction during the retirement transition by using multiple linear regression analyses with generalized estimating equations (GEEs). The GEE model controls for the intra-individual correlation between measurements waves, as one participant may contribute to multiple waves of life satisfaction measurements within the sleep problem group (Zeger et al. 1988; Diggle 2013). We defined the retirement transition period which covered wave − 1 to wave + 1 (on average 0.5 years before and 0.5 years after retirement), and for the analyses measuring the changes during the retirement transition (wave − 1 to wave + 1), the interaction term ‘sleep problem group × time (wave − 1 to wave + 1)’ was added to the GEE models. The covariates were included in the models across the whole retirement transition − 1 to + 1, as time-varying covariates. The estimates are presented as mean changes and their 95% CIs. The estimates and *p* values are given for the comparison of the other sleep problem groups with the ‘Never’ and ‘Increasing’ groups. The effect size is presented as standardized mean difference (SMD), which is the ratio of mean change and standard deviation (SD) (SMD = mean change/SD) (Cohen 2013; Andrade 2020).

The analyses of changes and baseline level of total and domain-specific life satisfaction are presented in three different models. The estimates presented in model 1 were adjusted for socio-demographic factors (sex, age, occupational status, and marital status), model 2 was additionally adjusted for lifestyle factors (body mass index, physical activity, and smoking), and model 3 was further adjusted for life events. All statistical analyses were conducted using the SAS Statistical Package version 9.4 (SAS Institute).

## Results

Characteristics of the participants before retirement are shown in Table 1. The average age of the study population was 63.4 years (SD 1.4), and the majority were women (83%). The distribution of study participants was almost equal in each of the three occupational status groups as there were 34% upper-grade nonmanual workers, 31% lower-grade nonmanual workers, and 35% manual workers.

**Table 1:** Descriptive statistics of the participants (*n* = 3518) at the pre-retirement wave − 1^a^ by sleep problem group^b^

Characteristics	Sleep problem group										
	All (*n* = 3518)		Never (*n* = 2225, 63%)		Decreasing (*n* = 397, 11%)		Increasing (*n* = 313, 9%)		Constant (*n* = 583, 17%)		*p* value^c^
	*m* (SD)/*n* (%)		*m* (SD)/*n* (%)		*m* (SD)/*n* (%)		*m* (SD)/*n* (%)		*m* (SD)/*n* (%)		
Age	63.4	(1.4)	63.5	(1.5)	63.3	(1.4)	63.4	(1.4)	63.3	(1.3)	0.193
Sex											0.080
Women	2927	(83)	1827	(82)	345	(87)	261	(83)	494	(85)	
Men	591	(17)	398	(18)	52	(13)	52	(17)	89	(15)	
Occupational status											0.205
Upper-grade nonmanual workers	1199	(34)	777	(35)	138	(35)	109	(35)	175	(30)	
Lower-grade nonmanual workers	1090	(31)	677	(30)	111	(28)	103	(33)	199	(34)	
Manual workers	1229	(35)	771	(35)	148	(37)	101	(32)	209	(36)	
Married/cohabited											0.507
Yes	2441	(71)	1549	(71)	276	(71)	226	(74)	390	(69)	
No	986	(29)	618	(29)	114	(29)	80	(26)	174	(31)	
Low physical activity											0.003
No	2192	(63)	1434	(65)	243	(61)	170	(55)	345	(60)	
Yes	1302	(37)	778	(35)	153	(39)	137	(45)	234	(40)	
Obesity											0.076
No	2730	(79)	1755	(80)	305	(78)	245	(79)	425	(75)	
Yes	747	(21)	450	(20)	86	(22)	66	(21)	145	(25)	
Current smoking											0.119
No	3149	(91)	1993	(91)	369	(94)	272	(89)	515	(90)	
Yes	305	(9)	195	(9)	23	(6)	32	(11)	55	(10)	
Life events (Wave + 1)											< 0.0001
No	2590	(74)	1695	(77)	283	(72)	222	(72)	390	(68)	
Yes	888	(26)	506	(23)	112	(28)	88	(28)	182	(32)	

^a^Wave − 1: 0.5 years before retirement. Note: The characteristics ‘Life events’ was measured at wave + 1: 0.5 year after retirement

^b^Sleep problem group: ‘Never’ (no sleep problems at wave − 1 nor at wave + 1), ‘Decreasing’ (sleep problems at wave − 1 but not at wave + 1), ‘Increasing’ (no sleep problems at wave − 1 but sleep problems at wave + 1), ‘Constant’ (sleep problems both at wave − 1 and wave + 1)

^c^*p* value based on χ^2^-test for categorical and ANOVA for continuous characteristics of the study population

There were almost no differences between the eligible population (*n* = 5195) and the survey population (*n* = 3518) in the beginning of the study regarding sex, age and occupational status (Online Supplementary Table 2).

A majority (63%) of the participants belonged to the ‘Never’ sleep problem group, followed by ‘Constant’ (17%), ‘Decreasing’ (11%), and the ‘Increasing’ sleep problems group (9%), respectively. The detailed description of the participants according to the sleep problem group at the pre-retirement wave − 1 is provided in Table 1. Briefly, when compared with the other sleep problem groups, the ‘Never’ group included a lower percentage of those who had experienced some negative life event during past year. Also, a higher proportion of those who did not have low physical activity belonged to ‘Never’ sleep problem group instead of ‘Increasing’ or ‘Constant’ group. The descriptive characteristics of the covariates according to the level of life satisfaction at pre-retirement wave − 1 are presented in Online Supplementary Table 3.

The total life satisfaction (*p* < 0.001) and the specific domains of life satisfaction (*p* < 0.05) before retirement (at wave − 1) differed by sleep problem groups so that the ‘Never’ group had higher total and domain-specific life satisfaction scores than the other sleep problem groups in the fully adjusted model (Online Supplementary Tables 4–5).

**Fig. 1:**
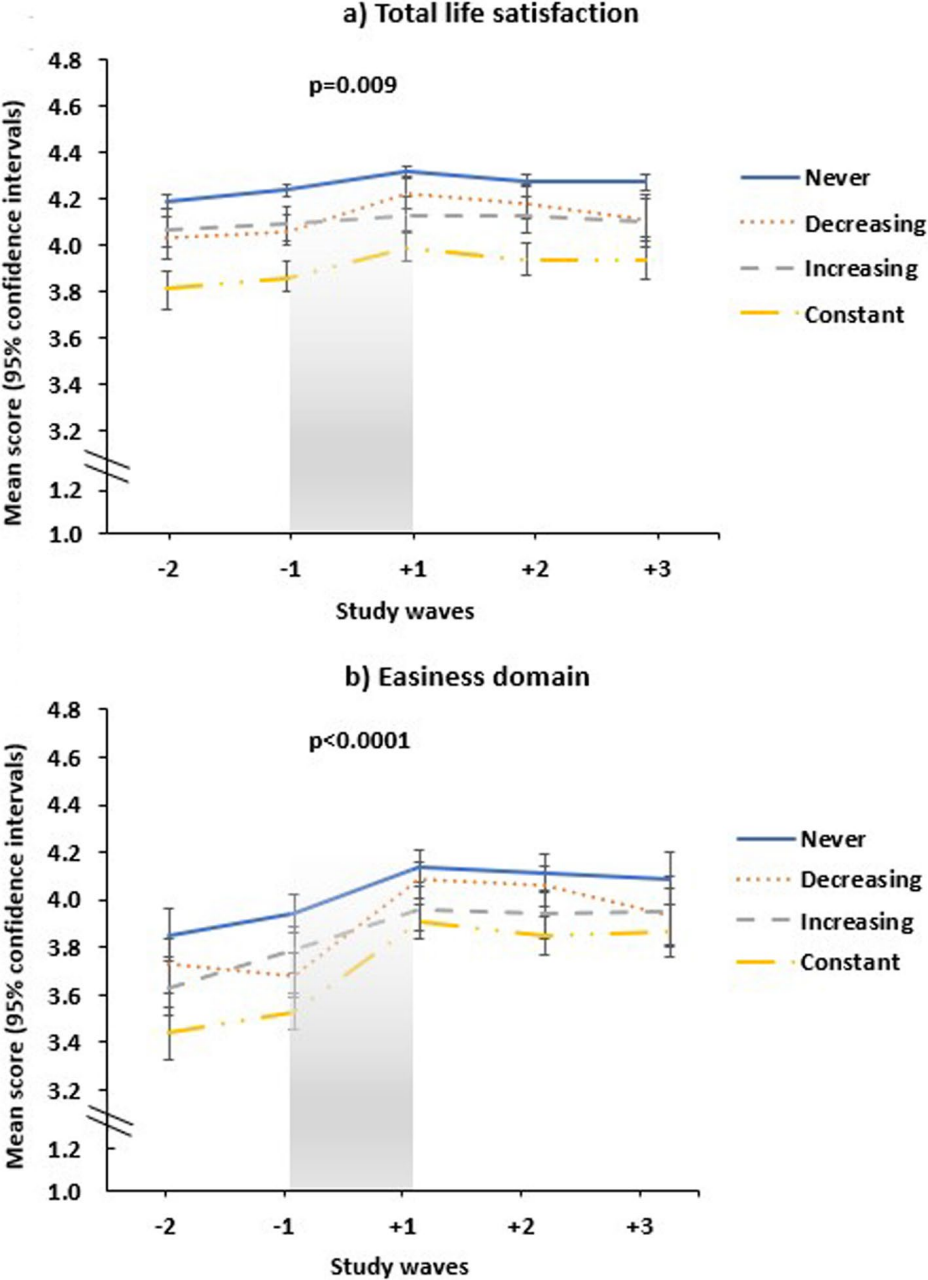
Mean scores (1–5) (and their 95% confidence intervals) for **a** total life satisfaction and **b** easiness domain between sleep problem groups. Note: ‘Never’ indicates participants without sleep problems during retirement transition, neither at wave − 1 nor at wave + 1, ‘Decreasing’ indicates participants having sleep problems at wave − 1, but not at wave + 1. ‘Increasing’ indicates participants not having sleep problems at wave − 1 but having at wave + 1. ‘Constant’ indicates participants having sleep problems during retirement transition, both at waves − 1 and + 1. Study waves describes the waves before retirement (− 2 to − 1), during retirement transition (− 1 to + 1) and after retirement (+ 1 to + 3). The unadjusted *p* values are for a differences between the sleep problem groups in changes in life satisfaction score during retirement transition period (wave − 1 to wave + 1)

Figure 1a illustrates the level of total life satisfaction in sleep problem groups before, during, and after retirement (wave − 2 to wave + 3). The level of total life satisfaction was highest in ‘Never’ group and lowest in ‘Constant’ group before, during, and after retirement. There was a significant difference in the increase in total life satisfaction between the sleep problem groups across the retirement transition (wave − 1 to wave + 1) (*p* = 0.009, sleep problem group × time interaction) when unadjusted. The total life satisfaction mean changes (mean chance estimates and their 95% CIs) during the retirement transition (wave − 1 to wave + 1) and standardized mean differences (SMD) for changes are presented in Table 2. Total life satisfaction increased slightly in all the other sleep problem groups during the retirement transition (SMD = 0.12–0.27) except in the ‘Increasing’ group. Total life satisfaction increased more among participants in the ‘Decreasing’ group than in the ‘Never’ group (*p* = 0.003) or in the ‘Increasing’ group (*p* = 0.002) according to the fully adjusted model. Total life satisfaction also increased more among participants in the ‘Constant’ group than among those in the ‘Increasing’ group (*p* = 0.037).

**Table 2:** Changes in total life satisfaction scores (mean change and its 95% CIs) during the retirement transition period (wave − 1 to wave + 1) by sleep problem group of the study population

	Model 1^a^			Model 2^b^			Model 3^c^			
	Mean change estimate (95% CI)	*p* value^e^ for difference with		Mean change estimate (95% CI)	*p* value^e^ for difference with		Mean change estimate (95% CI)	*p* value^e^ for difference with		SMD
		‘Never’ group	‘Increasing’ group		‘Never’ group	‘Increasing’ group		‘Never’ group	‘Increasing’ group	
Total	0.09 (0.07, 0.11)			0.09 (0.07, 0.11)			0.09 (0.07, 0.11)			0.14
Sleep problem group^d^										
Never	0.08 (0.05, 0.10)	Ref	0.258	0.08 (0.05, 0.10)	Ref	0.210	0.08 (0.05, 0.10)	Ref	0.202	0.12
Decreasing	0.16 (0.10, 0.22)	0.009	0.006	0.17 (0.11, 0.23)	0.004	0.003	0.17 (0.11, 0.23)	0.003	0.002	0.27
Increasing	0.04 (− 0.02, 0.11)	0.258	Ref	0.03 (− 0.04, 0.10)	0.210	Ref	0.03 (− 0.04, 0.10)	0.202	Ref	0.05
Constant	0.13 (0.08, 0.19)	0.076	0.033	0.12 (0.07, 0.18)	0.121	0.038	0.12 (0.07, 0.18)	0.123	0.037	0.19

*CI* confidence interval, *SMD* standardized mean difference

Wave − 1: 0.5 years before retirement, wave + 1: 0.5 years after retirement

^a^Model adjusted for age, gender, occupational status and marital status

^b^Model additionally adjusted for physical activity, BMI, and smoking

^c^Model additionally adjusted for life events

^d^Sleep problem group: ‘Never’ (no sleep problems at wave − 1 nor at wave + 1), ‘Decreasing’ (sleep problems at wave − 1 but not at wave + 1), ‘Increasing’ (no sleep problems at wave − 1 but sleep problems at wave + 1), ‘Constant’ (sleep problems both at wave − 1 and wave + 1)

^e^*p* value is for the difference in mean estimates in comparison to the sleep problem group of ‘Never’ or ‘Increasing’

We also examined the domains of life satisfaction before and after retirement (wave − 2 to + 3) (see Fig. 1b, and Online Supplementary Fig. 1a–c). The level of all domains (interestingness, happiness, easiness, togetherness) were highest in the ‘Never’ group and lowest in the ‘Constant’ group before, during, and after retirement. There was a significant difference in the increase in easiness domain between the sleep problem groups across the retirement transition (wave − 1 to wave + 1) (*p* < 0.0001, sleep problem group × time interaction), but not for the other domains of life satisfaction when unadjusted. The domain-specific life satisfaction mean changes (mean change estimates and their 95% CIs) during the retirement transition (wave − 1 to wave + 1) and standardized mean differences (SMD) for changes are presented in Table 3. The improvement for the easiness domain was observed in all sleep problem groups, with small to medium effect sizes (SMD = 0.22–0.61). The improvement was greater for participants in the ‘Decreasing’ group (*p* < 0.001) and in the ‘Constant’ group (*p* = 0.002) than for the ‘Increasing’ group. The improvement for the easiness domain was also greater for participants in the ‘Decreasing’ group (*p* < 0.001) and the ‘Constant’ group (*p* < 0.001) than in the ‘Never’ group.

**Table 3:** Changes in domain-specific life satisfaction scores (mean change and its 95% CIs) during the retirement transition period (wave − 1 to wave + 1) by sleep problem group of the study population

	Model 1^a^			Model 2^b^			Model 3^c^			
	Mean change 95% CI	*p* value^e^ for difference with		Mean change 95% CI	*p* value^e^ for difference with		Mean change 95% CI	*p* value^e^ for difference with		SMD
		‘Never’ group	‘Increasing’ group		‘Never’ group	‘Increasing’ group		‘Never’ group	‘Increasing’ group	
*Interestingness*										
Total	0.03 (0.00, 0.06)			0.03 (0.01, 0.06)			0.04 (0.01, 0.06)			0.06
Sleep problem group^d^										
Never	0.03 (− 0.01, 0.06)	Ref	0.559	0.03 (− 0.01, 0.06)	Ref	0.496	0.03 (− 0.00, 0.06)	Ref	0.440	0.05
Decreasing	0.07 (− 0.01, 0.15)	0.282	0.223	0.08 (0.00, 0.16)	0.206	0.155	0.08 (0.00, 0.16)	0.213	0.138	0.13
Increasing	− 0.00 (− 0.09, 0.09)	0.559	Ref	− 0.01 (− 0.10, 0.09)	0.496	Ref	− 0.01 (− 0.10, 0.08)	0.440	Ref	0.02
Constant	0.07 (− 0.01, 0.14)	0.319	0.246	0.07 (− 0.01, 0.14)	0.394	0.235	0.07 (− 0.01, 0.14)	0.382	0.220	0.11
*Happiness*										
Total	0.07 (0.05, 0.10)			0.07 (0.05, 0.10)			0.07 (0.05, 0.10)			0.11
Sleep problem group										
Never	0.07 (0.04, 0.10)	Ref	0.081	0.07 (0.04, 0.10)	Ref	0.049	0.07 (0.05, 0.10)	Ref	0.051	0.11
Decreasing	0.13 (0.06, 0.20)	0.107	0.012	0.13 (0.06, 0.20)	0.153	0.011	0.13 (0.06, 0.20)	0.140	0.010	0.20
Increasing	− 0.01 (− 0.09, 0.07)	0.081	Ref	− 0.02 (− 0.10, 0.07)	0.049	Ref	− 0.01 (− 0.10, 0.07)	0.051	Ref	0.02
Constant	0.10 (0.04, 0.16)	0.384	0.043	0.10 (0.03, 0.16)	0.479	0.036	0.10 (0.03, 0.16)	0.516	0.040	0.16
*Easiness*										
Total	0.24 (0.21, 0.27)			0.23 (0.20, 0.26)			0.23 (0.20, 0.26)			0.36
Sleep problem group										
Never	0.20 (0.16, 0.23)	Ref	0.419	0.19 (0.15, 0.23)	Ref	0.386	0.19 (0.15, 0.23)	Ref	0.393	0.30
Decreasing	0.38 (0.28, 0.48)	< 0.001	0.001	0.39 (0.28, 0.49)	< 0.001	0.001	0.39 (0.29, 0.49)	< 0.001	< 0.001	0.61
Increasing	0.15 0.05, 0.25)	0.419	Ref	0.14 (0.04, 0.25)	0.386	Ref	0.14 (0.04, 0.25)	0.393	Ref	0.22
Constant	0.37 (0.29, 0.45)	< 0.0001	0.001	0.36 (0.27, 0.44)	< 0.001	0.002	0.35 (0.27, 0.44)	< 0.001	0.002	0.55
*Togetherness*										
Total	0.02 (− 0.02, 0.06)		0.01 (− 0.03, 0.05)			0.01 (− 0.03, 0.05)			0.02	
Sleep problem group										
Never	0.03 (− 0.02, 0.07)	Ref	0.782	0.02 (− 0.03, 0.07)	Ref	0.741	0.02 (− 0.03, 0.07)	Ref	0.741	0.03
Decreasing	0.04 (− 0.06, 0.15)	0.748	0.650	0.05 (− 0.05, 0.16)	0.520	0.461	0.06 (− 0.05, 0.16)	0.509	0.453	0.09
Increasing	0.01 (− 0.10, 0.12)	0.782	Ref	− 0.00 (− 0.11, 0.11)	0.741	Ref	− 0.00 (− 0.11, 0.11)	0.741	Ref	0.00
Constant	− 0.01 (− 0.11, 0.09)	0.533	0.809	− 0.03 (− 0.13, 0.08)	0.424	0.739	− 0.02 (− 0.13, 0.08)	0.456	0.772	0.03

*CI* confidence interval, *SMD* standardized mean difference

Wave − 1: 0.5 years before retirement, wave + 1: 0.5 years after retirement

^a^Model adjusted for age, gender, occupational status and marital status

^b^Model additionally adjusted for physical activity, BMI, and smoking

^c^Model additionally adjusted for life events

^d^Sleep problem group: ‘Never’ (no sleep problems at wave − 1 nor at wave + 1), ‘Decreasing’ (sleep problems at wave − 1 but not at wave + 1), ‘Increasing’ (no sleep problems at wave − 1 but sleep problems at wave + 1), ‘Constant’ (sleep problems both at wave − 1 and wave + 1)

^e^*p* value is for the difference in mean estimates in comparison to the sleep problem group of ‘Never’ or ‘Increasing’

## Discussion

The current study is, to the best of our knowledge, the first study to examine concurrent changes in sleep problems and life satisfaction during the age-based retirement transition. We found that total life satisfaction increased most among those who had sleep problems before retirement and whose sleep problems relieved during the retirement transition, and among those who constantly had sleep problems. However, the effect sizes were modest. Similar patterns emerged in the easiness domain, whereas changes in sleep problems were not associated with changes in interestingness, happiness, or togetherness domains of life satisfaction.

One explanation for the findings of an improved total life satisfaction, and especially for the easiness domain, may be related to a removal of working hours from people’s daily schedules, leading to increased feelings of easiness. In addition, sleeping times can be arranged more freely, as people no longer have mandatory awakening times in the mornings. These changes can themselves reduce sleep problems and increase life satisfaction. On the other hand, living with constant sleep problems after retirement might be more manageable due to the freedom in scheduling, for example, it is possible to take naps. Additionally, the feeling of sleepiness is not as stressful in free time as when at work (Mullins et al. 2014).

We created four groups of sleep problems that described the condition of the participants’ sleep problems during the retirement transition (‘Never,’ ‘Decreasing,’ ‘Increasing,’ and ‘Constant’). When we compared these groups with each other, we found that the total life satisfaction and all the domains of life satisfaction before, during, and after the retirement transition were highest for those who never had sleep problems during the retirement transition, and lowest for those who constantly had sleep problems during the retirement transition. This is in correspondence with the previous studies showing that better sleep quality is associated with greater life satisfaction (Zhi et al. 2016; Ness and Saksvik-Lehouillier 2018; Papi and Cheraghi 2021).

Overall, life satisfaction was at a reasonably high level before retirement in terms of both the total and domain-specific life satisfaction in every sleep group. This may be led to marginal changes in life satisfaction during the retirement transition, with effect sizes ranging from small to medium. Additionally, previous Finnish studies on life satisfaction also reported notably high total life satisfaction levels (Koivumaa-Honkanen et al. 2000; Stenlund et al. 2021), although they did not consider sleep problems during the retirement transition. It is also notable, that the sleep problem groups with greatest improvements in terms of both total and domain-specific life satisfaction, namely ‘Decreasing’ and ‘Constant,’ had the lowest life satisfaction scores before retirement.

However, when assessing the significance of the results, many factors should be considered. For example, in large samples, even small and non-significant differences can be statistically significant (Andrade 2020). It has also been found that personality, genetic (Lyubomirsky et al. 2005), societal, social support, and income factors are the greatest contributors of subjective well-being leaving little space for the impact of other factors (Geerling and Diener 2020). Additionally, the effect sizes are best interpreted relative to the context of the current and prior studies (Henson 2006). Considering this study, it is difficult to define exactly what level of effect size would have a ‘real-world’ impact on life satisfaction in different sleep groups or what level of increase in life satisfaction score a person concretely notices in his/her life. However, taking into account previous studies, as well as the large sample size, numerous covariates, and moderate-level effect size this study suggests that the increased feeling of easiness, is a ‘real world’ experience after retirement for those whose sleep problems decreased or persisted at the retirement transition.

Our findings have theoretical and practical implications. Theoretically from a life satisfaction perspective, it is important to consider that the retirees may experience retirement transitions differently depending how their sleep problems develop. Our findings emphasize the importance of considering the interindividual variability of retirees’ sleep problems in a greater depth. Because life satisfaction during the retirement transition was associated with sleep problems in our study, and it has been associated with other individual (e.g., sex and self-rated health status), and contextual (e.g., spousal working status) factors in earlier studies as well (Prakash et al. 2022), this knowledge stresses the importance of studying life satisfaction during the retirement transition from a broader perspective, focusing on different individual and environmental characteristics of retirees (Wang 2007).

In relation to practical implications, sleep problems may decrease life satisfaction substantially (Paunio et al. 2008; Piper 2016). As our findings show, the retirees without sleep problems during the entire retirement transition had the highest total and domain-specific life satisfaction scores before, during, and after the retirement. Similarly, retirees experiencing constant sleep problems had the lowest life satisfaction throughout the retirement transition. However, life satisfaction increased most among those whose sleep problems relieved, and among those who had constant sleep problems during the retirement transition. This information may encourage employees to seek help for their sleep problems before retirement. Additionally, knowing that life can become easier despite sleep problems can bring relief to people of working-age who have sleep problems. Health professionals can use the findings to support the employees planning for retirement who would benefit from interventions to ease sleep problems. It should also be noted that although retirement itself seems to serve as an intervention to increase life satisfaction, life satisfaction can also be strengthened by various planned measures (Proyer et al. 2013; Mantelou and Karakasidou 2017), although their effectiveness has not been studied during the retirement transition or in the case of sleep problems.

The specific strengths of this study are a longitudinal study design and a considerably large cohort of older public sector employees, who were followed annually during their retirement transition. This type of repeated data enabled us to examine retirees’ intra-individual and concurrent changes in sleep and life satisfaction in retirement transition. The attrition analysis showed that the study population represents the eligible population very well. The availability of information on actual retirement age is also a strength of this study. When first contacted, all the respondents were still in employment. However, some of the respondents answered the annual survey for the first time only after retirement. It is also notable that this study did not only examine total life satisfaction but also domain-specific life satisfaction. As our findings suggest, by treating life satisfaction as a unidimensional phenomenon, we may lose important information on the specific domains. This is also confirmed by previous research (Bonsang and Klein 2012; Calasanti et al. 2021; Nakamura et al. 2022; Prakash et al. 2022).

The results of this study should be evaluated in the context of methodological limitations. First, all participant responses were collected via self-reports, which yields the possibility that the results may have been vulnerable to the effects of common-method bias (Podsakoff et al. 2003). The differential associations between life satisfaction domains and changes in sleep problems suggest that individual differences alone may not fully explain our findings. Life satisfaction represents internal psychological experiences and is most appropriately measured using self-reports provided by those who have directly experienced it. The participants were employed in the public sector in Finland and reached their statutory retirement age, and therefore, the findings may not necessarily be generalizable to those employed in other sectors and the general population. For example, study on seven European countries showed that public sector employees had higher levels of emotional well-being than private sector employees (Lahat and Ofek 2022). In addition, the results should be generalized cautiously to male employees, as 83% of our study population were women. However, this is in line with the statistics showing a similar proportion (78%) of public sector employees in Finland being women (Statistics Finland 2016). It is also possible that some other factors, for example depression, have affected the association between sleep problems and life satisfaction (Franzen and Buysse 2008; Nutt et al. 2008; Li et al. 2021; de Breij et al. 2022). However, it is important to notice that by studying sleep problems, we also studied one of the characteristics and diagnostic criteria of depression.

The study of life satisfaction will continue to be important because of the prevalence of chronic illnesses increases with advancing age, and as the life expectancy increases (Steptoe et al. 2015). Additionally, intergovernmental organizations [e.g., Organisation for Economic Co-operation and Development (OECD) and World Health Organization (WHO)] are urging the member countries to use well-being indicators, such as life satisfaction when making important policy decisions (Nakamura et al. 2022). Thus, future studies could examine the long-term association between life satisfaction and sleep problems after retirement and investigate whether improving sleep could support life satisfaction in post-retirement life. Since life satisfaction is a very subjective feeling, conducting in-depth interviews during the retirement transition could offer diverse insights into retirees experiences about the relationship between life satisfaction and sleep problems.

## Conclusions

The findings of this study suggest that decreasing or constant sleep problems are associated with improved life satisfaction during the retirement transition, especially in the feeling of easiness of life. It seems that the absence of working life schedule requirements during retirement improves life satisfaction by alleviating sleep problems or making it easier to live with them.

### Supplementary Information

Below is the link to the electronic supplementary material.

Supplementary file1 (DOCX 178 kb)

Supplementary file2 (DOCX 20 kb)

Supplementary file3 (DOCX 22 kb)

Supplementary file4 (DOCX 28 kb)

Supplementary file5 (DOCX 23 kb)

Supplementary file6 (DOCX 30 kb)

## Data Availability

Anonymized partial datasets of the Finnish Retirement and Aging Study are available by application with bona fide researchers with an established scientific record and bona fide organizations. For more information, please contact Prof. Sari Stenholm sari.stenholm[at]utu.fi.
