# Supplementary material for: Are changes in sleep problems associated with changes in life satisfaction during the retirement transition?
Source: Eur J Ageing. 2024 Mar 12;21(1):7. doi: 10.1007/s10433-024-00802-4 (PMC10933243; doi:10.1007/s10433-024-00802-4)
Supplement: Supplementary file 1 — Supplementary file1 (DOCX 178 kb) [file 10433_2024_802_MOESM1_ESM.docx]

**Are changes in sleep problems associated with changes in life satisfaction during the retirement transition?**

Marika Kontturi, MA^1*^, Marianna Virtanen, PhD^1,2^, Saana Myllyntausta, PhD^3^, Prakash KC, PhD^4^, Jaana Pentti, BSc^5,6,7^, Jussi Vahtera, PhD^5,7^, Sari Stenholm, PhD^5,7^

^1^School of Educational Sciences and Psychology, University of Eastern Finland, Joensuu, Finland

^2^Division of Insurance Medicine, Department of Clinical Neuroscience, Karolinska Institutet, Stockholm, Sweden

^3^Department of Psychology and Speech-Language Pathology, Faculty of Social Sciences, University of Turku, Turku, Finland

^4^Unit of Health Sciences, Faculty of Social Sciences, Tampere University, Tampere, Finland

^5^Department of Public Health, University of Turku and Turku University Hospital, Turku, Finland

^6^Clinicum, Faculty of Medicine, University of Helsinki, Helsinki, Finland

^7^Centre for Population Health Research, University of Turku and Turku University Hospital, Turku, Finland

*Corresponding author: Marika Kontturi ([marika.kontturi@uef.fi](mailto:marika.kontturi@uef.fi)), ORCID: 0000-0002-6245-4337

**SUPPLEMENTARY MATERIAL**


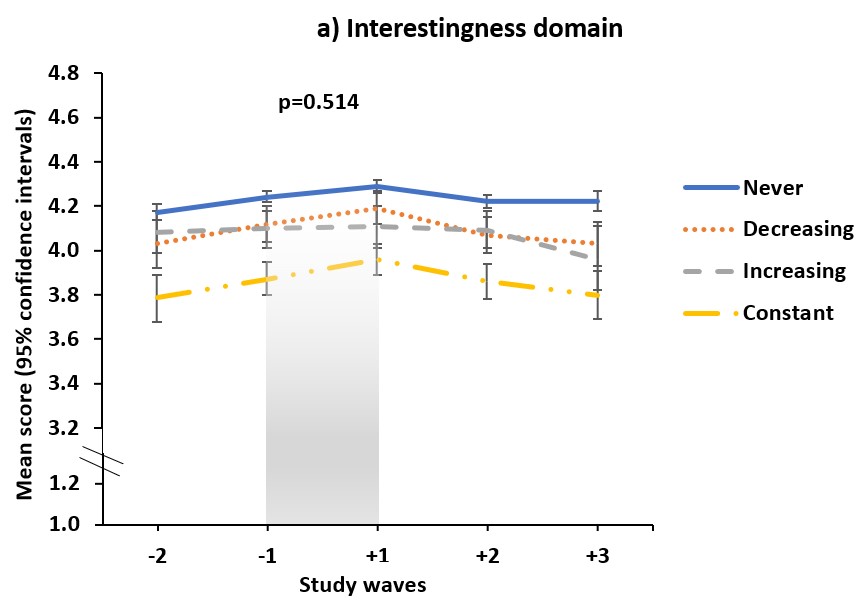


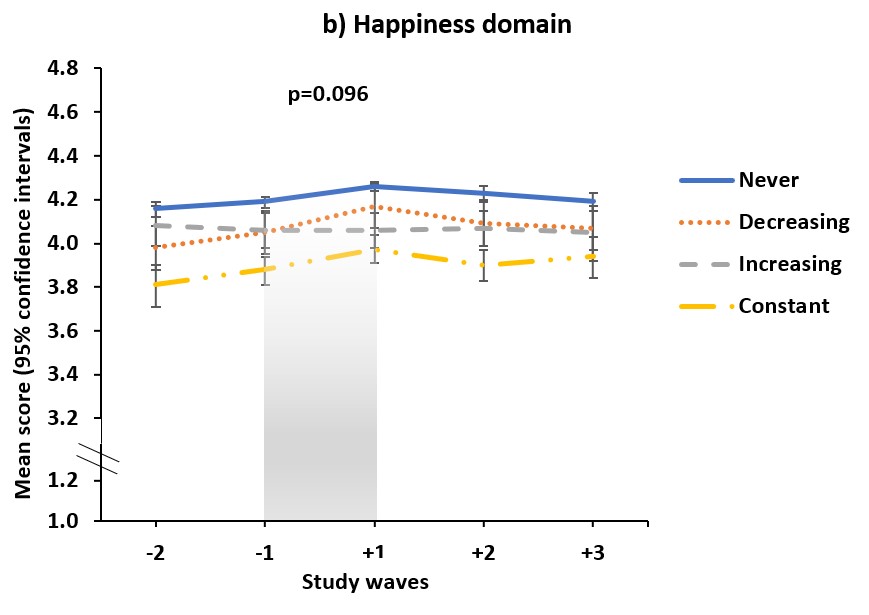


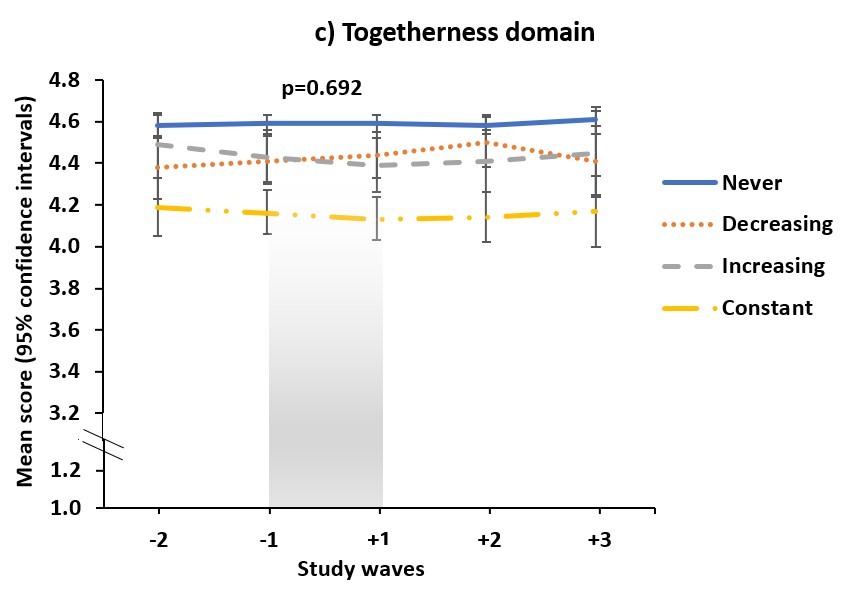


**Supplementary Fig SF1** Mean scores (1–5) (and their 95% confidence intervals) for a) interestingness domain, b) happiness domain, and c) togetherness domain between sleep problems groups

Note: ‘Never’ indicates participants without sleep problems during retirement transition, neither at wave -1 nor at wave +1, ‘Decreasing’ indicates participants having sleep problems at wave -1, but not at wave +1. ‘Increasing’ indicates participants not having sleep problems at wave -1 but having at wave +1. ‘Constant’ indicates participants having sleep problems during retirement transition, both at waves -1 and +1. Study waves describes the waves before retirement (-2 to -1), during retirement transition (-1 to +1) and after retirement (+1 to +3). The unadjusted p-values are for a differences between the sleep problem groups in changes in life satisfaction score during retirement transition period (wave -1 to wave +1).
