# Supplementary material for: Are changes in sleep problems associated with changes in life satisfaction during the retirement transition?
Source: Eur J Ageing. 2024 Mar 12;21(1):7. doi: 10.1007/s10433-024-00802-4 (PMC10933243; doi:10.1007/s10433-024-00802-4)
Supplement: Supplementary file 2 — Supplementary file2 (DOCX 20 kb) [file 10433_2024_802_MOESM2_ESM.docx]

**Are changes in sleep problems associated with changes in life satisfaction during the retirement transition?**

Marika Kontturi, MA^1*^, Marianna Virtanen, PhD^1,2^, Saana Myllyntausta, PhD^3^, Prakash KC, PhD^4^, Jaana Pentti, BSc^5,6,7^, Jussi Vahtera, PhD^5,7^, Sari Stenholm, PhD^5,7^

^1^School of Educational Sciences and Psychology, University of Eastern Finland, Joensuu, Finland

^2^Division of Insurance Medicine, Department of Clinical Neuroscience, Karolinska Institutet, Stockholm, Sweden

^3^Department of Psychology and Speech-Language Pathology, Faculty of Social Sciences, University of Turku, Turku, Finland

^4^Unit of Health Sciences, Faculty of Social Sciences, Tampere University, Tampere, Finland

^5^Department of Public Health, University of Turku and Turku University Hospital, Turku, Finland

^6^Clinicum, Faculty of Medicine, University of Helsinki, Helsinki, Finland

^7^Centre for Population Health Research, University of Turku and Turku University Hospital, Turku, Finland

*Corresponding author: Marika Kontturi ([marika.kontturi@uef.fi](mailto:marika.kontturi@uef.fi)), ORCID: 0000-0002-6245-4337

**SUPPLEMENTARY MATERIAL**

**Supplementary Table ST1** The study design

| **Pre-retirement period** | **Retirement transition period** | | | **Post-retirement period** | |
| --- | --- | --- | --- | --- | --- |
|  |  | Retirement |  |  |  |
| Wave -2  (n=2146) | Wave -1  (n=3518) |  | Wave +1  (n=3518) | Wave +2  (n=2823) | Wave +3  (n=1428) |
|  |  |  |  |  |  |

Waves around retirement period and the classification of pre-retirement period, retirement transition and post-retirement period of the study.
