# Supplementary material for: Are changes in sleep problems associated with changes in life satisfaction during the retirement transition?
Source: Eur J Ageing. 2024 Mar 12;21(1):7. doi: 10.1007/s10433-024-00802-4 (PMC10933243; doi:10.1007/s10433-024-00802-4)
Supplement: Supplementary file 3 — Supplementary file3 (DOCX 22 kb) [file 10433_2024_802_MOESM3_ESM.docx]

**Are changes in sleep problems associated with changes in life satisfaction during the retirement transition?**

Marika Kontturi, MA^1*^, Marianna Virtanen, PhD^1,2^, Saana Myllyntausta, PhD^3^, Prakash KC, PhD^4^, Jaana Pentti, BSc^5,6,7^, Jussi Vahtera, PhD^5,7^, Sari Stenholm, PhD^5,7^

^1^School of Educational Sciences and Psychology, University of Eastern Finland, Joensuu, Finland

^2^Division of Insurance Medicine, Department of Clinical Neuroscience, Karolinska Institutet, Stockholm, Sweden

^3^Department of Psychology and Speech-Language Pathology, Faculty of Social Sciences, University of Turku, Turku, Finland

^4^Unit of Health Sciences, Faculty of Social Sciences, Tampere University, Tampere, Finland

^5^Department of Public Health, University of Turku and Turku University Hospital, Turku, Finland

^6^Clinicum, Faculty of Medicine, University of Helsinki, Helsinki, Finland

^7^Centre for Population Health Research, University of Turku and Turku University Hospital, Turku, Finland

*Corresponding author: Marika Kontturi ([marika.kontturi@uef.fi](mailto:marika.kontturi@uef.fi)), ORCID: 0000-0002-6245-4337

**SUPPLEMENTARY MATERIAL**

**Supplementary Table ST2** Differences between the study population and the FIREA study eligible population and survey respondents

|  |  | | |  | |
| --- | --- | --- | --- | --- | --- |
|  | Eligible  population | Responded  at least once | Responded at least once  while at work | | Study  population |
| Characteristics* | (n=10629) | (n=6783) | (n=5195) | | (n=3518) |
| Age, mean (SD) | 62.7 (1.2) | 62.7 (1.2) | 62.5 (1.2) | | 62.5 (1.2) |
| Sex, % |  |  |  | |  |
| Women | 80 | 82 | 82 | | 83 |
| Men | 20 | 18 | 18 | | 17 |
| Occupational status, % |  |  |  | |  |
| Upper-grade nonmanual workers | 29 | 30 | 32 | | 34 |
| Lower-grade nonmanual workers | 29 | 30 | 31 | | 31 |
| Manual workers | 42 | 40 | 37 | | 35 |
| *At the beginning of the study | | | | | |
