# Supplementary material for: Are changes in sleep problems associated with changes in life satisfaction during the retirement transition?
Source: Eur J Ageing. 2024 Mar 12;21(1):7. doi: 10.1007/s10433-024-00802-4 (PMC10933243; doi:10.1007/s10433-024-00802-4)
Supplement: Supplementary file 4 — Supplementary file4 (DOCX 28 kb) [file 10433_2024_802_MOESM4_ESM.docx]

**Are changes in sleep problems associated with changes in life satisfaction during the retirement transition?**

Marika Kontturi, MA^1*^, Marianna Virtanen, PhD^1,2^, Saana Myllyntausta, PhD^3^, Prakash KC, PhD^4^, Jaana Pentti, BSc^5,6,7^, Jussi Vahtera, PhD^5,7^, Sari Stenholm, PhD^5,7^

^1^School of Educational Sciences and Psychology, University of Eastern Finland, Joensuu, Finland

^2^Division of Insurance Medicine, Department of Clinical Neuroscience, Karolinska Institutet, Stockholm, Sweden

^3^Department of Psychology and Speech-Language Pathology, Faculty of Social Sciences, University of Turku, Turku, Finland

^4^Unit of Health Sciences, Faculty of Social Sciences, Tampere University, Tampere, Finland

^5^Department of Public Health, University of Turku and Turku University Hospital, Turku, Finland

^6^Clinicum, Faculty of Medicine, University of Helsinki, Helsinki, Finland

^7^Centre for Population Health Research, University of Turku and Turku University Hospital, Turku, Finland

*Corresponding author: Marika Kontturi ([marika.kontturi@uef.fi](mailto:marika.kontturi@uef.fi)), ORCID: 0000-0002-6245-4337

**SUPPLEMENTARY MATERIAL**

**Supplementary Table ST3** Descriptive statistics of the participants (n=3518) at the pre-retirement wave -1^a^ by total and domain-specific life satisfaction at the wave -1

|  | Total life satisfaction | | | Life satisfaction domains | | | | | | | | | | | |
| --- | --- | --- | --- | --- | --- | --- | --- | --- | --- | --- | --- | --- | --- | --- | --- |
|  |  | | | Interesting | | | Happiness | | | Easiness | | | Togetherness | | |
| **Characteristics** | m(SD)/n(%) | | p-value^b^ | m(SD)/n(%) | | p-value^b^ | m(SD)/n(%) | | p-value^b^ | m(SD)/n(%) | | p-value^b^ | m(SD)/n(%) | | p-value^b^ |
| **Total (n=3518)** | 4.14 (0.64) | |  | 4.15 (0.75) | |  | 4.11 (0.65) | |  | 3.83 (0.84) | |  | 4.48 (1.09) | |  |
| Age |  |  | 0.095 |  |  | <0.0001 |  |  | 0.084 |  |  | 0.002 |  |  | 0.008 |
| <63 years (n=779)^c^ | 4.11 (0.62) | |  | 4.07 (0.73) | |  | 4.08 (0.64) | |  | 3.75 (0.88) | |  | 4.56 (1.02) | |  |
| ≥63 years (n=2739) | 4.15 (0.64) | |  | 4.18 (0.76) | |  | 4.12 (0.65) | |  | 3.85 (0.83) | |  | 4.46 (1.11) | |  |
| Sex |  |  | 0.176 |  |  | 0.619 |  |  | 0.446 |  |  | <0.001 |  |  | 0.841 |
| Women | 4.14 (0.64) | |  | 4.16 (0.76) | |  | 4.11 (0.66) | |  | 3.80 (0.86) | |  | 4.48 (1.19) | |  |
| Men | 4.18 (0.60) | |  | 4.14 (0.73) | |  | 4.13 (0.61) | |  | 3.94 (0.78) | |  | 4.49 (1.10) | |  |
| Occupational status |  |  | <0.001 |  |  | <0.0001 |  |  | <0.0001 |  |  | 0.393 |  |  | 0.053 |
| Upper-grade nonmanual workers | 4.19 (0.62) | |  | 4.26 (0.74) | |  | 4.17 (0.63) | |  | 3.83 (0.86) | |  | 4.51 (1.07) | |  |
| Lower-grade nonmanual workers | 4.09 (0.66) | |  | 4.08 (0.77) | |  | 4.05 (0.66) | |  | 3.80 (0.86) | |  | 4.41 (1.15) | |  |
| Manual workers | 4.15 (0.64) | |  | 4.13 (0.74) | |  | 4.11 (0.66) | |  | 3.85 (0.82) | |  | 4.51 (1.06) | |  |
| Married/cohabited |  |  | <0.0001 |  |  | <0.0001 |  |  | <0.0001 |  |  | 0.001 |  |  | <0.0001 |
| Yes | 4.22 (0.57) | |  | 4.20 (0.72) | |  | 4.16 (0.62) | |  | 3.86 (0.82) | |  | 4.66 (0.90) | |  |
| No | 3.96 (0.74) | |  | 4.06 (0.83) | |  | 3.99 (0.71) | |  | 3.76 (0.89) | |  | 4.03 (1.36) | |  |
| Low physical activity |  |  | <0.0001 |  |  | <0.0001 |  |  | <0.0001 |  |  | <0.0001 |  |  | 0.002 |
| No | 4.19 (0.59) | |  | 4.21 (0.70) | |  | 4.15 (0.61) | |  | 3.88 (0.81) | |  | 4.53 (1.05) | |  |
| Yes | 4.06 (0.71) | |  | 4.06 (0.83) | |  | 4.05 (0.70) | |  | 3.74 (0.90) | |  | 4.41 (1.16) | |  |
| Obesity |  |  | 0.079 |  |  | 0.020 |  |  | 0.318 |  |  | 0.283 |  |  | 0.235 |
| No | 4.16 (0.62) | |  | 4.17 (0.74) | |  | 4.12 (0.64) | |  | 3.84 (0.83) | |  | 4.50 (1.07) | |  |
| Yes | 4.11 (0.67) | |  | 4.10 (0.80) | |  | 4.09 (0.69) | |  | 3.80 (0.87) | |  | 4.45 (1.14) | |  |
| Current smoking |  |  | <0.0001 |  |  | <0.0001 |  |  | <0.0001 |  |  | 0.259 |  |  | <0.0001 |
| No | 4.16 (0.62) | |  | 4.18 0.73 | |  | 4.13 (0.64) | |  | 3.83 (0.84) | |  | 4.51 (1.07) | |  |
| Yes | 3.95 (0.77) | |  | 3.85 0.92 | |  | 3.91 (0.77) | |  | 3.78 (0.87) | |  | 4.24 (1.28) | |  |
| Life events (Wave +1) |  |  | <0.0001 |  |  | <0.0001 |  |  | <0.0001 |  |  | <0.0001 |  |  | <0.0001 |
| No | 4.16 (0.62) | |  | 4.20 (0.71) | |  | 4.16 (0.61) | |  | 3.90 (0.79) | |  | 4.54 (1.03) | |  |
| Yes | 3.95 (0.77) | |  | 4.02 (0.87) | |  | 3.97 (0.74) | |  | 3.62 (0.95) | |  | 4.31 (1.23) | |  |

^a^Wave -1: 0.5 years before retirement. Note: The characteristics ‘Life events’ was measured at the wave +1: 0.5 years after retirement.

^b^A t-test was used to examine the association between dichotomized categorical characteristics and life satisfaction.

An analysis of variance was used to examine the association between categorical characteristic (>2 levels = occupational status) and life satisfaction.

A Pearson correlation coefficient was used to examine the association between continuous characteristic (age) and life satisfaction.

^c^determining the mean (SD) of participants under 63 years, and participants 63 years and older. 63 years was the median value of age of participants at the pre-retirement wave -1.
