# Supplementary material for: Are changes in sleep problems associated with changes in life satisfaction during the retirement transition?
Source: Eur J Ageing. 2024 Mar 12;21(1):7. doi: 10.1007/s10433-024-00802-4 (PMC10933243; doi:10.1007/s10433-024-00802-4)
Supplement: Supplementary file 5 — Supplementary file5 (DOCX 23 kb) [file 10433_2024_802_MOESM5_ESM.docx]

**Are changes in sleep problems associated with changes in life satisfaction during the retirement transition?**

Marika Kontturi, MA^1*^, Marianna Virtanen, PhD^1,2^, Saana Myllyntausta, PhD^3^, Prakash KC, PhD^4^, Jaana Pentti, BSc^5,6,7^, Jussi Vahtera, PhD^5,7^, Sari Stenholm, PhD^5,7^

^1^School of Educational Sciences and Psychology, University of Eastern Finland, Joensuu, Finland

^2^Division of Insurance Medicine, Department of Clinical Neuroscience, Karolinska Institutet, Stockholm, Sweden

^3^Department of Psychology and Speech-Language Pathology, Faculty of Social Sciences, University of Turku, Turku, Finland

^4^Unit of Health Sciences, Faculty of Social Sciences, Tampere University, Tampere, Finland

^5^Department of Public Health, University of Turku and Turku University Hospital, Turku, Finland

^6^Clinicum, Faculty of Medicine, University of Helsinki, Helsinki, Finland

^7^Centre for Population Health Research, University of Turku and Turku University Hospital, Turku, Finland

*Corresponding author: Marika Kontturi ([marika.kontturi@uef.fi](mailto:marika.kontturi@uef.fi)), ORCID: 0000-0002-6245-4337

**SUPPLEMENTARY MATERIAL**

**Supplementary Table ST4** Mean estimates and their 95% CIs for difference in total life satisfaction score before retirement (wave -1) by sleep problem group of the study population

|  |  | Model 1^a^ | | |  | Model 2^b^ | | |  | Model 3^c^ | | |
| --- | --- | --- | --- | --- | --- | --- | --- | --- | --- | --- | --- | --- |
|  |  | Mean estimate  (95% CI) | | p-value^e^ for difference to ’Never’ group |  | Mean estimate  (95% CI) | | p-value^e^ for difference to ’Never’ group |  | Mean estimate  (95% CI) | | p-value^e^ for difference to ’Never’ group |
| **Total** |  | 4.08 | (4.05, 4.11) |  |  | 4.00 | (3.96, 4.05) |  |  | 3.96 | (3.91, 4.00) |  |
| **Sleep problem group^d^** |  |  |  |  |  |  |  |  |  |  |  |  |
| Never |  | 4.18 | (4.14, 4.21) | Ref. |  | 4.09 | (4.05, 4.14) | Ref. |  | 4.05 | (4.00, 4.10) | Ref. |
| Decreasing |  | 4.00 | (3.94, 4.07) | <0.0001 |  | 3.91 | (3.84, 3.99) | <0.0001 |  | 3.88 | (3.80, 3.95) | <0.0001 |
| Increasing |  | 4.03 | (3.95, 4.10) | <0.0001 |  | 3.96 | (3.88, 4.04) | <0.001 |  | 3.92 | (3.84, 4.00) | <0.001 |
| Constant |  | 3.81 | (3.76, 3.86) | <0.0001 |  | 3.74 | (3.68, 3.81) | <0.0001 |  | 3.71 | (3.65, 3.78) | <0.0001 |

CI: Confidence interval

-1: 0.5 years before retirement

^a^Model adjusted for age, gender, occupational status, and marital status

^b^Model additionally adjusted for physical activity, BMI, and smoking

^c^Model additionally adjusted for life events

^d^Sleep problem group: ‘Never’ (no sleep problems at wave -1 nor at wave +1), ‘Decreasing’ (sleep problems at wave -1 but not at wave +1), ‘Increasing’ (no sleep problems at wave -1 but sleep problems at wave +1), ‘Constant’ (sleep problems both at wave -1 and wave +1)

^e^p-value is for the difference in mean estimates in comparison to the sleep problem group of ‘Never’
