# Supplementary material for: Are changes in sleep problems associated with changes in life satisfaction during the retirement transition?
Source: Eur J Ageing. 2024 Mar 12;21(1):7. doi: 10.1007/s10433-024-00802-4 (PMC10933243; doi:10.1007/s10433-024-00802-4)
Supplement: Supplementary file 6 — Supplementary file6 (DOCX 30 kb) [file 10433_2024_802_MOESM6_ESM.docx]

**Are changes in sleep problems associated with changes in life satisfaction during the retirement transition?**

Marika Kontturi, MA^1*^, Marianna Virtanen, PhD^1,2^, Saana Myllyntausta, PhD^3^, Prakash KC, PhD^4^, Jaana Pentti, BSc^5,6,7^, Jussi Vahtera, PhD^5,7^, Sari Stenholm, PhD^5,7^

^1^School of Educational Sciences and Psychology, University of Eastern Finland, Joensuu, Finland

^2^Division of Insurance Medicine, Department of Clinical Neuroscience, Karolinska Institutet, Stockholm, Sweden

^3^Department of Psychology and Speech-Language Pathology, Faculty of Social Sciences, University of Turku, Turku, Finland

^4^Unit of Health Sciences, Faculty of Social Sciences, Tampere University, Tampere, Finland

^5^Department of Public Health, University of Turku and Turku University Hospital, Turku, Finland

^6^Clinicum, Faculty of Medicine, University of Helsinki, Helsinki, Finland

^7^Centre for Population Health Research, University of Turku and Turku University Hospital, Turku, Finland

*Corresponding author: Marika Kontturi ([marika.kontturi@uef.fi](mailto:marika.kontturi@uef.fi)), ORCID: 0000-0002-6245-4337

**SUPPLEMENTARY MATERIAL**

**Supplementary Table ST5** Mean estimates and their 95% CIs for difference in domain-specific life satisfaction score before retirement (wave -1) by sleep problem group of the study population

|  |  |  | Model 1^a^ | | |  | Model 2^b^ | | |  | Model 3^c^ | | |
| --- | --- | --- | --- | --- | --- | --- | --- | --- | --- | --- | --- | --- | --- |
|  |  |  | Mean estimate (95% CI) | | p-value^e^ for difference to ‘Never’ group |  | Mean estimate  (95% CI) | | p-value^e^ for difference to ‘Never’ group |  | Mean estimate  (95% CI) | | p-value^e^ for difference to ‘Never’ group |
| **Interestingness** | **Total** |  | 4.10 | (4.07, 4.14) |  |  | 3.97 | (3.91, 4.02) |  |  | 3.93 | (3.87, 3.98) |  |
|  | **Sleep problem group^d^** |  |  |  |  |  |  |  |  |  |  |  |  |
|  | Never |  | 4.19 | (4.15, 4.23) | Ref. |  | 4.05 | (4.00, 4.11) | Ref. |  | 4.02 | (3.96, 4.08) | Ref. |
|  | Decreasing |  | 4.06 | (3.98, 4.14) | 0.002 |  | 3.92 | (3.83, 4.00) | 0.001 |  | 3.88 | (3.79, 3.97) | 0.002 |
|  | Increasing |  | 4.03 | (3.95, 4.12) | <0.001 |  | 3.91 | (3.82, 4.01) | 0.002 |  | 3.89 | (3.79, 3.98) | 0.003 |
|  | Constant |  | 3.83 | (3.77, 3.90) | <0.0001 |  | 3.71 | (3.63, 3.78) | <0.0001 |  | 3.68 | (3.60, 3.76) | <0.0001 |
| **Happiness** | **Total** |  | 4.07 | (4.04, 4.10) |  |  | 3.99 | (3.94, 4.03) |  |  | 3.95 | (3.90, 4.00) |  |
|  | **Sleep problem group** |  |  |  |  |  |  |  |  |  |  |  |  |
|  | Never |  | 4.14 | (4.11, 4.18) | Ref. |  | 4.06 | (4.01, 4.11) | Ref. |  | 4.02 | (3.97, 4.07) | Ref. |
|  | Decreasing |  | 4.01 | (3.94, 4.07) | <0.0001 |  | 3.93 | (3.85, 4.00) | <0.001 |  | 3.89 | (3.81, 3.97) | <0.001 |
|  | Increasing |  | 4.01 | (3.94, 4.08) | <0.001 |  | 3.94 | (3.86, 4.03) | 0.002 |  | 3.91 | (3.83, 3.99) | 0.004 |
|  | Constant |  | 3.85 | (3.79, 3.90) | <0.0001 |  | 3.78 | (3.71, 3.84) | <0.0001 |  | 3.75 | (3.68, 3.82) | <0.0001 |
| **Easiness** | **Total** |  | 3.85 | (3.81, 3.89) |  |  | 3.82 | (3.76, 3.88) |  |  | 3.77 | (3.71, 3.83) |  |
|  | **Sleep problem group** |  |  |  |  |  |  |  |  |  |  |  |  |
|  | Never |  | 3.95 | (3.91, 4.00) | Ref. |  | 3.92 | (3.86, 3.99) | Ref. |  | 3.87 | (3.80, 3.93) | Ref. |
|  | Decreasing |  | 3.71 | (3.62, 3.80) | <0.0001 |  | 3.68 | (3.58, 3.78) | <0.0001 |  | 3.63 | (3.53, 3.73) | <0.0001 |
|  | Increasing |  | 3.82 | (3.72, 3.92) | 0.008 |  | 3.81 | (3.70, 3.92) | 0.026 |  | 3.76 | (3.66, 3.87) | 0.039 |
|  | Constant |  | 3.55 | (3.47, 3.62) | <0.0001 |  | 3.54 | (3.45, 3.62) | <0.0001 |  | 3.50 | (3.41, 3.59) | <0.0001 |
| **Togetherness** | **Total** |  | 4.32 | (4.26, 4.37) |  |  | 4.23 | (4.16, 4.31) |  |  | 4.19 | (4.11, 4.27) |  |
|  | **Sleep problem group** |  |  |  |  |  |  |  |  |  |  |  |  |
|  | Never |  | 4.42 | (4.36, 4.48) | Ref. |  | 4.34 | (4.26, 4.42) | Ref. |  | 4.29 | (4.21, 4.38) | Ref. |
|  | Decreasing |  | 4.24 | (4.13, 4.35) | 0.002 |  | 4.15 | (4.02, 4.28) | 0.002 |  | 4.11 | (3.98, 4.24) | 0.002 |
|  | Increasing |  | 4.25 | (4.12, 4.37) | 0.006 |  | 4.17 | (4.03, 4.30) | 0.009 |  | 4.12 | (3.99, 4.26) | 0.008 |
|  | Constant |  | 4.00 | (3.91, 4.10) | <0.0001 |  | 3.94 | (3.83, 4.05) | <0.0001 |  | 3.91 | (3.80, 4.02) | <0.0001 |

CI: Confidence Interval

Wave -1: 0.5 years before retirement

^a^Model adjusted for age, gender, occupational status, and marital status

^b^Model additionally adjusted for physical activity, BMI, and smoking

^c^Model additionally adjusted for life events

^d^Sleep problem group: ‘Never’ (no sleep problems at wave -1 nor at wave +1), ‘Decreasing’ (sleep problems at wave -1 but not at wave +1), ‘Increasing’ (no sleep problems at wave -1 but sleep problems at wave +1), ‘Constant’ (sleep problems both at wave -1 and wave +1)

^e^p-value is for the difference in mean estimates in comparison to the sleep problem group of ‘Never’
